# ^18^F-FDG PET/CT Findings in Glandular Tularemia

**DOI:** 10.3390/diagnostics15091159

**Published:** 2025-05-02

**Authors:** Freja Gustafsson, Karl Keigo Rasmussen, Kristina Thorsteinsson, Anne-Mette Lebech, Lasse Fjordside

**Affiliations:** 1 Department of Infectious Diseases, Copenhagen University Hospital—Rigshospitalet, Blegdamsvej 9, DK-2100 Copenhagen, Denmark; 2 Department of Nuclear Medicine, Copenhagen University Hospital—Bispebjerg, Bispebjerg Bakke 23, DK-2400 Copenhagen, Denmark; 3 Department of Pulmonary and Infectious Diseases, Copenhagen University Hospital—Bispebjerg, Bispebjerg Bakke 23, DK-2400 Copenhagen, Denmark; 4 Department of Clinical Medicine, University of Copenhagen, Blegdamsvej 3B, DK-2200 Copenhagen, Denmark

**Keywords:** ^18^F-FDG PET/CT, *Francisella tularensis*, Tularemia

## Abstract

A 47-year-old woman presented with fever, fatigue, night sweats and inguinal glandular swelling following a tick bite. Weeks of diagnostic uncertainty followed, and a lymph node biopsy was sent to be investigated for tularemia and pathology. An ^18^F-FDG PET/CT scan was performed due to a suspicion of malignant lymphoma. The scan revealed high metabolic activity in the left inguinal region, which was compatible with abscesses. The diagnosis of glandular tularemia was established on a positive PCR for *Francisella tularensis* (*F. tularensis*) and positive *F. tularensis* serology. This case highlights the challenges of diagnosing tularemia and illustrates the role of imaging.

**Figure 1:**
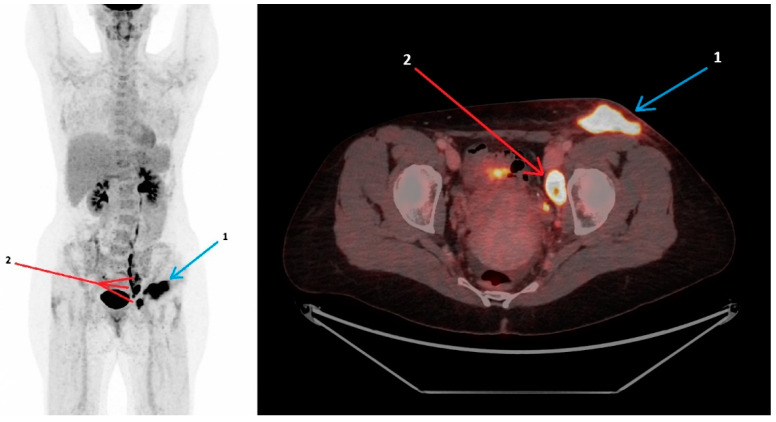
A 47-year-old woman, previously healthy, presented in July 2024 with pain in the left groin, fever and general malaise following a tick bite five days earlier. An ultrasound scan was performed, revealing enlarged inguinal lymph nodes. The laboratory testing showed elevated C-reactive protein (CRP) at 22 mg/L (normal range 0–10 mg/L). Four days later, the patient developed night sweats and worsening of the pain, and the CRP was measured to 84 mg/L. Treatment with oral penicillin V (1200 mg three times daily for a total of 10 days) was initiated upon suspicion of Lyme borreliosis (Erythema migrans). Three weeks later, the patient was readmitted due to the progression of symptoms, with growing glandular swelling, new-onset inguinal redness, fatigue and continued night sweats. In the meantime, treatment with dicloxacillin for seven days was attempted, due to a suspicion of erysipelas, without improvement. An ultrasound scan showed increased sizes of reactive lymph nodes, and repeated blood work revealed a lower but still elevated CRP at 28 mg/L. A lymph node biopsy was performed and sent for culture, pathology and PCR for *Francisella tularensis* (*F. tularensis*) and *Mycobacterium tuberculosis.* The patient then initiated empiric treatment with doxycycline. Additional diagnostic work-up included serology (HIV, syphilis, cytomegalovirus, Epstein–Barr virus, toxoplasmosis, tick-borne encephalitis virus, bartonella, quantiferon) and blood cultures. All came out negative. The prolonged course of illness, the presence of B-symptoms, the lack of response to the initial antibiotic treatment, and the diagnostic uncertainty raised concerns of a malignant etiology, prompting an ^18^F-FDG PET/CT scan. The patient was scanned on a GE Discovery MI 5 ring PET/CT scanner. The scan included a diagnostic CT with contrast enhancement using Omnipaque (350 mg I/mL), with a 110 mL intravenous injection. The PET tracer F-18-FDG 199 MBq was administered by intravenous injection approximately one hour before acquisition. The scan (Figure 1) showed a maximal intensity projection and axial fused ^18^F-FDG PET/CT with high metabolic activity in the subcutaneous phlegmone/abscess in the left inguinal region (arrow 1), with stranding in the surrounding subcutaneous fat and high metabolic activity in enlarged lymph nodes along the left iliacal vessels, one of which had an internal abscess (arrow 2). The abscess measured 4.3 × 2.4 cm, with an SUV_max_ of 14.5, and the largest lymph node measured 3.2 × 1.9 cm. The SUV_max_ of the iliacal lymph nodes was 14.3 (for reference, the liver SUV_max_ was 4.0). The diagnosis of glandular tularemia was established when the PCR for *F. tularensis* on the lymph node biopsy that was performed earlier came out positive alongside a positive *F. tularensis* serology. The ^18^F-FDG PET/CT and clinical manifestation corroborated this finding. PCR and cultivation for *Mycobacterium tuberculosis* from the lymph nodes were both negative. The patient continued doxycycline 200 mg daily for a total of four weeks, resulting in clinical improvement and CRP normalization. A follow-up ultrasound scan performed after completed treatment revealed resolution of the abscess and regression of lymphadenopathy [1]. Tularemia is a zoonotic infection caused by the Gram-negative bacteria *F. tularensis*. The transmission routes of *F. tularensis* include contact with tissues of infected animals, inhalation of aerosols, consumption of contaminated water or food and arthropod bites, e.g., by ticks [2,3]. An overall increase in cases of tick-borne diseases, including tularemia, has been reported, emphasizing the importance of clinical awareness to ensure timely diagnosis and treatment [4]. The clinical presentation of tularemia varies depending on the route of transmission, virulence and patient’s immune status. The wide spectrum of clinical presentations can complicate the diagnostic process, potentially resulting in treatment delays. Manifestations include ulcero-glandular, glandular, oro-pharyngeal, pneumonic, typhoidal and oculo-glandular tularemia [3,5]. The diagnosis of tularemia is based on serology with the identification of specific *F. tularensis* antibodies in serum or detection of *F. tularensis* DNA using PCR in relevant samples [5]. Consequently, ^18^F-FDG PET/CT is not part of the standard diagnostic work-up. However, given the significant variety of clinical manifestations and the non-specific symptoms seen in tularemia, several differential diagnoses may be relevant, and ^18^F-FDG PET/CT can be a valuable tool for discriminating between malignant and inflammatory conditions, as reported in this case. To the best of our knowledge, no other data on ^18^F-FDG-PET/CT findings in glandular tularemia have been reported. However, a few published cases describe ^18^F-FDG PET/CT scan findings in pneumonic tularemia. Similarly to the presented case, the scans were performed due to suspicion of malignancy, with pneumonic tularemia mimicking lung cancer. Scans revealed a high FDG uptake in lymph nodes and pulmonary lesions that were compatible with pneumonic tularemia; however, they were not specific to the disease. These cases illustrate the diagnostic challenges of tularemia and conclude that pneumonic tularemia is an important differential diagnosis to lung cancer in endemic areas. Furthermore, they demonstrate that exposure history, as well as the presence of infectious symptoms, is crucial in targeting the diagnosis of tularemia [6,7]. The presented case also highlights the diagnostic challenges of tularemia, whose symptoms may mimic malignancy. Our case suggests that ^18^F-FDG PET/CT can be a useful tool in similar cases to aid the decision to target diagnostic efforts at infectious etiologies. With scan findings, as in the presented case, showing glandular inflammation correlated with a history of tick exposure, clinicians should consider tularemia as a potential diagnosis.

## Data Availability

Not applicable.
